# Traversing missing links in the spread of HIV

**DOI:** 10.7554/eLife.82610

**Published:** 2022-09-30

**Authors:** Erin Brintnell, Art Poon

**Affiliations:** 1 Department of Pathology and Laboratory Medicine, Western University London Canada; 2 Department of Computer Science, Western University London Canada

**Keywords:** HIV-1, phylogenetics, transmission chains, molecular epidemiology, Viruses

## Abstract

Combining clinical and genetic data can improve the effectiveness of virus tracking with the aim of reducing the number of HIV cases by 2030.

**Related research article** Blenkinsop A, Monod M, van Sighem A, Pantazis N, Bezemer D, Op de Coul E, van de Laar T, Fraser C, Prins M, Reiss P, de Bree GJ, Ratmann O. 2022. Estimating the potential to prevent locally acquired HIV infections in a UNAIDS fast-track City, Amsterdam. *eLife* **11**:e76487. doi: 10.7554/eLife.76487.

The human immunodeficiency virus type 1 (HIV-1), which can lead to acquired immune deficiency syndrome (AIDS), remains a leading cause of death and a health threat worldwide, with over 38.4 million people currently living with the virus. Global health sector strategies strive to end HIV-1 epidemics by 2030 (Duncombe et al., 2019). This requires significant investment in resources to treat and prevent the disease, such as reducing the number of people who do not know they are carrying the virus and improving the availability and affordability of effective treatments.

In cities that have scaled up HIV-1 treatment and prevention, it will be crucial to establish whether new HIV-1 infections are due to ongoing local transmission or to infections acquired abroad. This means reconstructing the spread of HIV-1 between individuals through contact tracing: interviewing people recently diagnosed with HIV, and locating and notifying their intimate partners. However, contact tracing is both time-consuming and intrusive (El-Sadr et al., 2022).

A cost-effective alternative to contact tracing is to compare the genomic sequences of HIV-1 samples from different patients, which are often collected to screen for mutations that confer drug resistance. Infections that are genetically similar are more likely to be related through recent transmissions. This is especially true for HIV-1, a rapidly evolving virus that becomes genetically unique within months of an infection (Williamson, 2003).

These genetic sequences can be used to build a tree that represents the shared evolutionary history of the infections and approximates the history of recent transmissions (De Maio et al., 2018; Romero-Severson et al., 2014). Furthermore, the spread of infections from one place to another can be extrapolated by reconstructing locations of ‘ancestral’ infections at deeper nodes of the tree from the known locations at the tips (Faria et al., 2011). The accuracy of these estimates, however, is impeded by the unknown number of people with undiagnosed infections, or with diagnosed infections that have not been sequenced (Didelot et al., 2017). In addition, reconstructing transmission patterns from HIV-1 sequences comes with its own ethical challenges because HIV-1 transmission is criminalized in many countries (Dawson et al., 2020).

Now, in eLife, Oliver Ratmann at the HIV Transmission Elimination Amsterdam Consortium and colleagues – including Alexandra Blenkinsop as first author – report an innovative approach to overcome the disadvantages of sequence analysis (Blenkinsop et al., 2022). Blenkinsop et al. combined different data sources to reconstruct the transmission histories of HIV-1 in Amsterdam, which has the highest concentration of HIV-1 cases in the Netherlands. Amsterdam is also part of the Fast-Track city network, which provides funds to expand effective HIV prevention, testing and treatment services.

Blenkinsop et al. extended the standard approach of reconstructing transmission histories from HIV-1 sequences by incorporating additional information from clinical biomarkers (biological indicators of disease progression or response to treatment) and other patient data (Figure 1). A statistical model was fitted to two biomarkers: the number of HIV-1 particles circulating in the blood (the viral load) and the number of white blood cells targeted by HIV-1. Based on how these biomarkers changed over time, it was possible to estimate the length of time between a person’s HIV-1 infection and diagnosis. These estimates were then used to infer how many cases were transmitted from people with unsequenced infections, adjusting for factors like route of transmission (e.g., injection drug use), age group, and place of birth.

**Figure 1.**
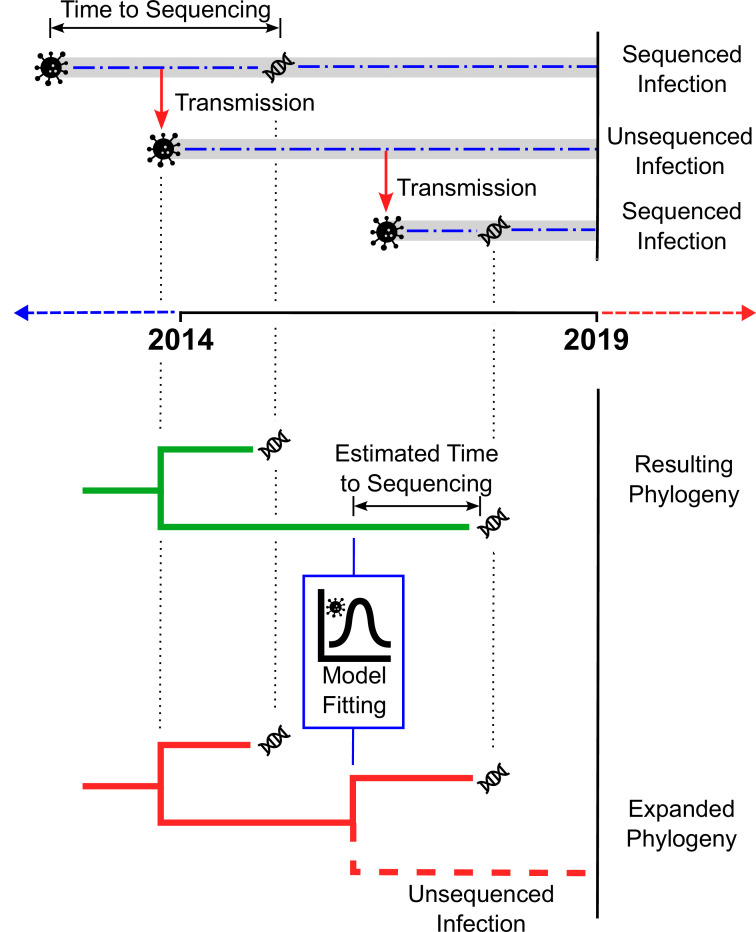
Estimating the number of unsampled HIV-1 infections. The top panel illustrates how a chain of HIV-1 infections may be partially sampled over time. The top dashed line shows an infection (represented by the virus particle symbol) that is transmitted (red arrow) before it is sequenced (DNA symbol), with the time between the infection occurring and sequencing taking place indicated by the two-headed arrow. The dashed line in the centre shows an infection resulting from transmission from the first infection, which is transmitted (red arrow) but never sequenced. The dashed line on the bottom represents a third infection resulting from the second infection, that is sequenced (DNA symbol) more quickly than the original infection. The bottom panel depicts two phylogenetic trees. The first tree (green) is inferred from the available sequences (in this case, the two infections sequenced in the top panel). By fitting a statistical model to HIV-1 cases with estimated dates of infection and clinical data, the number of unsampled infections (‘missing links’) in the new tree (red) can be extrapolated for different populations.

Despite extensive measures to curb the transmission of HIV in Amsterdam, results from Blenkinsop et al. suggest that many HIV-1 infections have remained undiagnosed for a long time, especially among heterosexual residents and recent arrivals from sub-Saharan Africa. Further, they provide evidence of ongoing HIV-1 transmission within the city over the duration of the five-year study. These results suggest that, while Amsterdam has made significant progress in reducing the spread of HIV-1, closing the final gap to end the local epidemic by 2030 remains a challenge.

The study also highlights the importance of linking HIV-1 sequences to both clinical and demographic information to determine which groups have been neglected by the generalized scale-up of public health testing and treatment. This may also be a critical step for other cities in the FastTrack initiative. Furthermore, the work of Blenkinsop et al. mirrors ongoing challenges in tracking and controlling other infectious diseases like COVID-19, which is characterised by an abundance of viral genome sequences but a lack of linked contextual information, including clinical outcomes, travel histories and sampling strategies (Chiara et al., 2021; Chen et al., 2022).
